# Psychological Impact of COVID‐19 on Nurses and Midwives in Albania: Fear, Anxiety and Predictors of Emotional Burden

**DOI:** 10.1002/nop2.71001

**Published:** 2026-11-07

**Authors:** Anila Cake, Ergys Ramosaçaj, Artan Simaku, Agron Bytyqi, Flora Zyberaj

**Affiliations:** ^1^ Faculty of Technical Medical Sciences – FSHMT University of Medicine Tirana Albania; ^2^ Institute of Public Health Tirana Albania; ^3^ Patients’ Rights Association in Kosovo, PRAK Prizren Kosovo

**Keywords:** Albania, COVID‐19, fear, midwives, nurses, psychological impact, trait anxiety

## Abstract

**Aim:**

To examine associations between recalled COVID‐19‐related emotional burden, demographic, health and occupational factors, psychological support and trait anxiety among nurses and midwives in Albania.

**Design:**

A quantitative cross‐sectional analytical study using retrospective assessments of pandemic‐related emotional experiences.

**Methods:**

Between March 2024 and May 2025, 1201 nurses and midwives from 17 public healthcare institutions in Albania completed structured questionnaires. Stratified random sampling was used. Descriptive analyses included the full cohort, while multivariable linear regression was conducted among 677 participants with complete trait anxiety and predictor data.

**Results:**

Mean recalled anxiety, fear and distress scores were 6.53, 6.77 and 7.04, respectively, on 0–10 scales. Overall, 80.6% believed psychological support would have improved their coping. Fear showed the strongest emotional association with trait anxiety, followed by worry. Chronic disease and worsened perceived quality of life were associated with higher trait anxiety, whereas male gender, older age, greater professional experience and reported psychological support showed inverse associations. The adjusted regression model explained 55% of the variance in trait anxiety.

**Conclusion:**

Recalled pandemic‐related fear and worry were associated with trait anxiety among nurses and midwives. Findings should be interpreted cautiously because of the retrospective assessment, cross‐sectional design, and complete‐case analysis.

**Implications for the Profession and/or Patient Care:**

Nursing leadership should prioritize accessible, confidential psychological support and integrate workforce mental health into emergency preparedness strategies.

**Impact:**

This study identifies emotional and health‐related factors associated with anxiety in the Albanian nursing and midwifery workforce, informing occupational health planning and future intervention research.

**Reporting Method:**

The study was reported in accordance with the Strengthening the Reporting of Observational Studies in Epidemiology (STROBE) guidelines.

**No Patient or Public Contribution:**

The study involved nurses and midwives as research participants. Patients and members of the public were not involved in the study design, conduct, analysis or reporting.

## 1 Introduction

While COVID‐19 has been widely characterized as a physical health emergency, its psychological impact on healthcare workers represents a parallel, often‐overlooked crisis. Frontline healthcare professionals—particularly nurses and midwives—faced sustained emotional pressure arising from prolonged patient exposure, limited protective resources, high workloads, fear of transmitting infection to family members, and societal stigma (Shanafelt et al. 2020; Lai et al. 2020).

Nurses and midwives form the backbone of hospital and community care systems globally. In Albania, they constitute a large share of the healthcare workforce, yet evidence remains limited on how pandemic‐specific fear, worry, health vulnerability, and access to psychological support jointly relate to anxiety in these professional groups. This gap is especially relevant to nursing science because nurses and midwives combine sustained patient contact with staffing, family, and organizational pressures that can shape both workforce wellbeing and continuity of care.

Fear has emerged as a significant psychological stressor linked to mental health deterioration during pandemics, with elevated fear associated with anxiety and other forms of psychological distress among frontline workers (Brooks et al. 2020). Reports from nursing populations in Iran similarly described substantial psychological strain during COVID‐19 (Kamran, Malekpour, and Naeim 2021; Kamran, Naeim, and Rezaeisharif 2021). Intolerance of uncertainty—difficulty adapting to unpredictable situations—has also been identified as an important vulnerability construct during crisis periods.

Additional evidence directly involving nurses and midwives strengthens the context for this study. Aksoy and Kocak (2020) assessed anxiety and intolerance of uncertainty in 758 nurses and midwives during the acute outbreak in Turkey. Kamran, Malekpour, and Naeim (2021) provide a brief contextual account of psychological burden among Iranian nurses. More recently, Rahmani et al. (2024) examined psychological distress and work‐related factors in relation to coping among 365 nurses, highlighting the relevance of occupational context alongside individual vulnerability.

The intervention and recommendation literature should be distinguished. Naeim et al. (2021) reported improvements in anxiety, stress, and fatigue after virtual cognitive‐behavioural group therapy in a small controlled pretest–post‐test study of 40 intensive care nurses, described as quasi‐experimental. Its small single‐centre sample limits generalization. The publications by Naeim et al. (2020) and Kamran, Naeim, and Bagvand (2020) offer strategies and recommendations rather than controlled evidence of effectiveness. Kamran, Naeim, and Rezaeisharif (2020) provide broader commentary on pandemic‐related psychological responses and public perceptions. These sources support the rationale for investigating staff burden and support needs without establishing intervention effects in the present cohort.

The specific research gap addressed here is the absence of Albanian evidence quantifying the independent associations of recalled pandemic‐related fear and worry with trait anxiety among nurses and midwives while simultaneously accounting for health, demographic, occupational, and psychological‐support factors. A related publication from the same dataset has since characterized post‐pandemic state/trait anxiety, intolerance of uncertainty, chronic conditions, and professional experience using a different analytical model (Cake et al. 2026). The present report addresses a distinct question by focusing on COVID‐19‐specific emotional responses and a different complete‐case multivariable model.

Accordingly, this study aimed to examine: (1) the association between self‐reported pandemic‐related fear and STAI‐T trait anxiety; (2) associations of intolerance of uncertainty with emotional outcomes; (3) the contribution of emotional, demographic, health, and occupational factors to trait anxiety; and (4) perceived and reported psychological support in relation to emotional burden.

## 2 Methods

### 2.1 Study Design and Participants

A quantitative cross‐sectional analytical study was conducted from March 2024 to May 2025 among nurses and midwives employed in 17 public healthcare institutions in Albania, including university, regional, and municipal services across different geographic areas. Data collection therefore occurred after the World Health Organization ended the COVID‐19 public health emergency of international concern on 5 May 2023; pandemic‐related emotional items were reported retrospectively during this post‐emergency period (World Health Organization 2023).

The post‐emergency assessment addresses remembered pandemic experiences in relation to anxiety proneness relevant to workforce support in 2024–2025. It was not a prospective follow‐up of individuals assessed during the acute outbreak. No pre‐pandemic STAI‐T baseline or contemporaneous pandemic‐emotion assessment was available for within‐person comparison. Therefore, the design cannot reconstruct acute‐period symptom prevalence or demonstrate persistence of symptoms within individual participants.

Reporting of this cross‐sectional observational study follows the STROBE recommendations.

An item‐by‐item STROBE checklist is supplied as Supplementary File 1, including the limitations of the available recruitment and analytical documentation.

According to national workforce statistics available to the study team, the eligible professional population comprised 26,490 nurses, 2413 midwives, and 2100 professionals qualified as both nurses and midwives (total 31,003). The sampling frame consisted of institutional staff lists or official ward‐level rosters of eligible nurses and midwives supplied by participating institutions.

Strata were defined by professional role (nurse or midwife) and institution/hospital type. Where feasible, proportional allocation was used so that invitations from each stratum reflected the approximate distribution of eligible staff within participating institutions. Eligible individuals were then selected randomly from staff lists or ward rosters within each stratum. Replacement was permitted only when a selected individual was ineligible, unavailable during data collection, or declined participation. This operational procedure is the basis for the term stratified random sampling used in this report.

The sample size calculation assumed a 95% confidence level, an expected proportion of 50% (*p* = 0.50), and a 3% margin of error, with an allowance for anticipated nonresponse. A total of 1201 questionnaires met the predefined completeness/quality criteria and formed the descriptive study cohort. Because a standardized centralized log of every invitation, refusal, and non‐return was not maintained across all 17 institutions, an exact response proportion cannot be reported.

Inclusion criteria were: (1) registered nurse or midwife actively employed in a participating public healthcare institution; (2) at least 6 months of professional experience in the current role; (3) age 18 years or older; and (4) ability to complete the questionnaire in Albanian. Individuals on long‐term medical, administrative, or maternity leave during data collection, and questionnaires with substantial missing or internally inconsistent data, were excluded from the descriptive dataset.

### 2.2 Instruments and Data Collection

Participants completed an anonymous, self‐administered structured questionnaire adapted from the survey framework used by Aksoy and Kocak (2020). The Albanian version underwent linguistic and conceptual review by the research team and pilot testing before implementation. The questionnaire contained the following components.

#### 2.2.1 Sociodemographic and Professional Data

Age, gender, marital status, education level, years of professional experience, professional role (nurse vs. midwife), institution type, department assignment, and presence of chronic health conditions.

#### 2.2.2 Trait Anxiety

Measured using the Trait form of the State‐Trait Anxiety Inventory (STAI‐T; Spielberger et al. 1983). The STAI‐T is a validated, widely used instrument assessing chronic anxiety proneness independent of current emotional state. Scores range from 20 to 80, with higher scores indicating greater trait anxiety.

Trait anxiety concerns a relatively enduring propensity to experience anxiety, whereas the retrospective pandemic‐specific items refer to remembered experiences in a particular context. STAI‐T was selected to characterize anxiety proneness at survey completion, not to establish a pre‐pandemic trait baseline. Trait anxiety is not immutable and may share negative‐affect content with recalled emotions. Treating emotions as statistical predictors does not establish causal or temporal precedence: current anxiety may influence the recall of prior fear and worry.

#### 2.2.3 Emotional Impact of COVID‐19

Single 0–10 rating items captured recalled pandemic‐related fear, anxiety, distress, worry, and surprise. These items were treated as individual self‐report indicators and not as a separately validated multi‐item psychometric scale.

These numerical ratings were study‐specific single‐item indicators within the adapted survey framework. Questionnaire review and pilot testing support comprehensibility but do not establish psychometric validation of individual items. Test–retest reliability, convergent validity, and measurement equivalence between nurses and midwives were not established. The items were not summed into a diagnostic emotional‐burden scale, and no clinical cut‐off was applied.

#### 2.2.4 Intolerance of Uncertainty

The 12‐item Intolerance of Uncertainty Scale (IUS‐12; Carleton et al. 2007) measured discomfort with unpredictable and ambiguous situations. Scores range from 12 to 48, with higher scores indicating greater intolerance of uncertainty.

#### 2.2.5 Psychological Support

Questionnaire items recorded whether participants reported receiving psychological support and whether they believed such support during the pandemic would have helped them cope better; perceived potential effectiveness was rated on a 0–10 scale.

#### 2.2.6 Quality of Life

Participants reported whether they perceived their life quality to have worsened during the pandemic (yes/no).

The final questionnaire was distributed in printed format. Selected staff were approached within participating institutions and completed the questionnaire independently during staff breaks or other non‐critical periods. Designated head nurses facilitated only logistical distribution and collection; they did not review individual responses. Participants received study information, provided written informed consent, and were assured that participation was voluntary, responses were anonymous, and nonparticipation would have no professional consequences.

Retrospective ratings may be influenced by selective memory, particularly salient events and respondents' current psychological state. Memory decay may attenuate reported intensity, while adverse experiences or current anxiety may intensify recollection. The direction and size of this bias cannot be quantified without contemporaneous measurements. Anonymity may reduce reporting pressure but does not eliminate recall or common‐method bias.

The full descriptive cohort comprised *N* = 1201. For the primary multivariable analysis, complete STAI‐T data and complete data for all emotional and sociodemographic predictors were available for *n* = 677 participants (56.4% of the descriptive cohort); 524 participants (43.6%) were therefore not included in that complete‐case regression.

### 2.3 Statistical Analysis

Data analysis was conducted using IBM SPSS Statistics version 25.0. Descriptive statistics (means, standard deviations, frequencies, and percentages) summarized participant characteristics and psychological measures. Denominators were based on valid responses available for each descriptive item.

Inferential analyses included independent samples *t*‐tests and one‐way analysis of variance (ANOVA) for group comparisons, with Bonferroni correction applied for multiple comparisons. Pearson correlation coefficients examined associations between continuous variables. Multicollinearity was assessed using variance inflation factors (VIF).

The primary multiple linear regression model included emotional predictors (fear, worry, distress, surprise) and sociodemographic/health variables (age, gender, marital status, reported work sector, years of experience, chronic disease, worsened life quality, and reported psychological support) to identify factors statistically associated with STAI‐T scores. Given the central role of fear as the construct of interest, a simpler fear‐focused sensitivity model was also examined.

The model was fitted by ordinary least squares with simultaneous entry of all 12 covariates. Fear, worry, distress, surprise, age, and years of professional experience were entered as continuous variables. Gender, marital status, work sector, chronic disease, worsened life quality and receipt of psychological support were represented by indicator variables. The corresponding reference categories were female gender, not currently married, private work sector, no chronic disease, no worsening of life quality, and no reported psychological support. Covariate selection reflected the study's emotional, demographic, occupational, and health framework rather than automated stepwise selection based on univariable *p*‐values. No interaction terms were fitted. Age and experience were retained as related but substantively distinct characteristics.

A two‐sided significance threshold of alpha = 0.05 was applied. The adjusted coefficient of determination (adjusted *R* ^2^) quantified model explanatory performance. The multivariable analysis used complete cases only (*n* = 677); no imputation was performed. Consequently, regression estimates describe the complete‐case analytic subset rather than all 1201 descriptive participants.

Participants included in the primary complete‐case regression (*n* = 677) were compared with those excluded (*n* = 524) to assess observed differences relevant to selection and generalizability. Age and professional experience were compared using two‐sided Welch independent‐samples *t*‐tests; binary characteristics were compared using Pearson chi‐squared tests without continuity correction. Standardized mean differences (SMDs), calculated as included minus excluded, described the magnitude and direction of between‐group differences. These descriptive selection comparisons were not adjusted for multiplicity and were not used to establish the missing‐data mechanism.

Additional comparisons used professional role, with nurses and midwives compared separately and two dual‐role participants omitted from that comparison. These additional analyses were conducted in Python with NumPy, pandas, and SciPy. For this comparison, the 20 adapted trait items were rescored by reversing positively worded items N1, N6, N7, N10, N13, N16, and N19 and requiring all 20 responses to be valid (1–4). Nonnumeric or out‐of‐range responses were treated as unavailable. This scoring definition applies specifically to the additional profession comparisons.

The added continuous comparisons use medians and interquartile ranges and two‐sided Mann–Whitney tests; categorical comparisons use Pearson chi‐squared tests. Holm adjustment covers nine comparisons. Mean differences are nurse minus midwife, with 95% confidence intervals clustered by the 20 recorded recruitment units. Distributional *p*‐values and confidence intervals for mean differences concern different estimands; the rank and chi‐square tests do not adjust for unit clustering. These analyses are exploratory and do not establish equivalence or causal professional differences.

Interpretation of a multiple linear regression requires distinguishing model fit, coefficient precision, and the assumptions governing inference. Adjusted *R* ^2^ summarizes apparent fit after accounting for model size; it is not an assessment of performance in an independent population (Altman and Royston 2000). VIF and tolerance concern dependence among predictors and should be interpreted alongside coefficient uncertainty and substantive overlap between variables, rather than by a numerical threshold alone (O'Brien 2007). They do not evaluate residual normality, linearity, constant residual variance, influential observations, or dependence among respondents within institutions.

Additional model diagnostics comprised the Shapiro–Wilk test of residual normality, the studentized Breusch–Pagan test for heteroscedasticity (Koenker 1981), and a Ramsey RESET specification test using squared and cubed fitted values. Externally studentized residuals, leverage, and Cook's distance were examined (Cook 1977). Screening thresholds were |studentized residual| > 3, leverage > 2(13)/677 = 0.0384, and Cook's distance > 4/677 = 0.00591. These thresholds identified observations for review, not automatic deletion. Sensitivity analyses used HC3 heteroscedasticity‐consistent standard errors (MacKinnon and White 1985), a refit excluding observations above the Cook's‐distance threshold, and the simpler fear‐only model. The primary analysis retained all 677 complete cases. The additional diagnostic calculations used Python, NumPy, and SciPy. HC3 inference addresses heteroscedasticity but does not account for within‐institution correlation.

### 2.4 Ethical Considerations

The study was conducted in accordance with the Declaration of Helsinki and received ethical approval from the Ethics Council of the University of Medicine, Tirana, Albania (protocol no. 825/2; decision no. 10; 18 March 2024), before data collection began. Institutional authorization was obtained from all 17 participating institutions. All participants received study information and provided written informed consent before participation. Participation was voluntary; questionnaires were anonymous, no direct identifiers were collected, and confidentiality was maintained. Consent for publication was not applicable because no identifiable individual‐level information is reported.

### 2.5 Relationship to Prior Publication

A related analysis of the same cross‐sectional dataset was published during the revision period (Cake et al. 2026). That article focused on post‐pandemic state and trait anxiety, intolerance of uncertainty, chronic conditions, and professional experience and used a different predictor set and analytic sample. The present manuscript focuses on recalled COVID‐19‐specific emotional responses (fear, worry, distress, and surprise), psychological‐support variables, and their associations with STAI‐T in the complete‐case subset of *n* = 677. The related publication is cited here to make the dataset overlap and analytical distinction explicit.

## 3 Results

### 3.1 Participant Characteristics

Table 1 presents the demographic and professional characteristics of the participants.

**TABLE 1:** Demographic and professional characteristics of participants (*N* = 1201).

Variable	*n* or Mean (SD)	% or Range
Gender		
Female	1023	85.2
Male	178	14.8
Age M (SD)	39.5 ± 10.9	21–66
Age group		
21–30	331	27.8
31–40	380	31.9
41–50	222	18.7
51–60	237	19.9
60+	20	1.7
Marital status		
Single	879	73.2
Married	320	26.6
Divorced/Widowed	2	0.2
Level of education		
BSc Nursing	452	37.6
BSc Midwifery–Nursing	4	0.3
BSc Midwifery	115	9.6
Other degree	22	1.8
MSc Nursing	512	42.6
MSc Midwifery	94	7.8
PhD Nursing	2	0.2
Area of residence		
Rural	123	10.2
Urban	1078	89.8

Demographic and professional characteristics of the study sample (*N* = 1201): The mean age was 39.5 ± 10.9 years, with 85.2% female participants. The majority (89.8%) resided in urban areas. Regarding professional composition, 80.0% were nurses and 19.8% were midwives. Professional experience ranged widely: 26.0% reported less than 5 years experience, 37.9% reported 6–15 years experience, and 36.1% reported more than 15 years experience. Chronic illness was reported by 22.7% of participants, with hypertension (7.2%), thyroid disorders (5.1%), and diabetes (4.8%) being most prevalent.

Analytic sample for the primary objective: Of the 1201 participants contributing to descriptive reporting, 677 (56.4%) had complete STAI‐T and predictor data and were included in the primary multivariable regression; 524 (43.6%) were excluded from that model because at least one required variable was incomplete. No missing values were imputed.

### 3.2 Emotional Burden and Psychological Outcomes

Participants with available COVID‐19 emotional‐impact data reported substantial recalled burden. Mean scores on the 0–10 items were 6.53 ± 1.87 for anxiety, 6.77 ± 1.92 for fear, and 7.04 ± 1.95 for distress. These 0–10 ratings are distinct from the STAI‐T instrument; STAI‐T was used as the dependent variable in the multivariable model reported below.

The Intolerance of Uncertainty Scale (IUS‐12) demonstrated positive correlations with all emotional distress indicators (*r* range = 0.41–0.47, *p* < 0.001), indicating that greater difficulty tolerating uncertainty was consistently associated with heightened emotional distress.

Notably, 80.6% of participants reported believing that psychological support during the pandemic would have assisted them in coping more effectively. When asked to rate the perceived effectiveness of psychological support, participants averaged 9.0 ± 0.8 on a 0–10 scale, indicating very high perceived potential benefit.

In subgroup analyses, emotional distress also differed significantly by chronic illness, age, gender, and professional experience (all *p* < 0.05), indicating that emotional burden was not uniform across participant subgroups.

### 3.3 Multivariate Regression Analysis: Predictors of Trait Anxiety

Table 2 presents the complete‐case multivariable linear regression model (*n* = 677) predicting STAI‐T scores from emotional and sociodemographic/health predictors. The overall model was statistically significant (*p* < 0.001), with adjusted *R* ^2^ = 0.55. These model results therefore pertain to the 677 participants with complete data for all included variables.

**TABLE 2:** Multivariable linear regression model predicting STAI‐T scores (complete‐case *n* = 677).

Predictor	*B*	SE(*B*)	Standardized beta	*t*	*p*	95% CI	VIF
Fear	1.48	0.14	0.34	10.7	< 0.001	[1.21, 1.75]	1.18
Worry	0.94	0.38	0.14	2.48	0.013	[0.20, 1.68]	1.22
Distress	0.37	0.32	0.06	1.17	0.242	[−0.25, 0.99]	1.15
Surprise	0.21	0.28	0.03	0.75	0.454	[−0.34, 0.76]	1.06
Gender (male)	−2.80	0.85	−0.13	−3.29	0.001	[−4.60, −1.00]	1.12
Age	−0.12	0.04	−0.10	−2.86	0.004	[−0.20, −0.04]	1.08
Marital status (married)	1.90	0.82	0.09	2.31	0.021	[0.30, 3.60]	1.14
Work sector (public)	3.50	1.12	0.15	3.12	0.002	[1.20, 5.80]	1.17
Years of work experience	−0.20	0.07	−0.12	−2.86	0.004	[−0.40, −0.05]	1.09
Chronic disease	4.10	1.05	0.18	3.90	< 0.001	[2.00, 6.20]	1.11
Worsened life quality	2.70	0.90	0.12	3.00	0.003	[1.00, 4.40]	1.13
Psychological support (received)	−2.10	0.82	−0.10	−2.56	0.011	[−3.70, −0.50]	1.10

*Note:* Model summary: *n* = 677; 12 predictors plus the intercept; residual df = 664; adjusted *R* ^2^ = 0.55; overall model *p* < 0.001. VIF range 1.06–1.22; corresponding tolerance range approximately 0.820–0.943.

Abbreviations: *B*, unstandardized coefficient; CI, confidence interval; SE, standard error; VIF, variance inflation factor.

#### 3.3.1 Model Interpretation

Fear emerged as the strongest emotional predictor of trait anxiety, accounting for the largest standardized effect (*β* = 0.34, *p* < 0.001). Each one‐point increase on the 0–10 fear scale was associated with a 1.48‐point increase in STAI‐T scores. Worry was the second‐most significant emotional predictor (*β* = 0.14, *p* = 0.013), though with a smaller effect magnitude. Distress and surprise, while included in the model, did not achieve statistical significance (*p* = 0.242 and *p* = 0.454, respectively). Their nonsignificance does not establish that these experiences were transient or clinically unimportant.

Among sociodemographic variables, chronic disease demonstrated the largest effect (*β* = 0.18, *p* < 0.001), with participants reporting chronic conditions exhibiting STAI‐T scores approximately 4.1 points higher than those without chronic illness. Worsened perceived life quality was the second‐strongest demographic predictor (*β* = 0.12, *p* = 0.003), associated with a 2.7‐point increase in STAI‐T scores.

Work sector showed a substantial effect: public sector employment was associated with 3.5‐point higher STAI‐T scores compared to private sector employment (*β* = 0.15, *p* = 0.002). Marital status was also significant: married participants exhibited 1.9‐point higher anxiety scores than unmarried participants (*β* = 0.09, *p* = 0.021).

Inverse associations with STAI‐T were observed for male gender (*β* = −0.13, *p* = 0.001), older age (beta = −0.10, *p* = 0.004), greater work experience (*β* = −0.12, *p* = 0.004), and reported psychological support (*β* = −0.10, *p* = 0.011). These coefficients are statistical associations in a cross‐sectional model and should not be interpreted as causal protective effects.

The inverse coefficients should be interpreted only as conditional associations. They do not demonstrate that support, age, experience, or gender causes lower anxiety. A nonsignificant emotional coefficient likewise does not establish that the corresponding experience was transient or clinically unimportant.

The primary model included 12 predictors and an intercept, with 677 complete cases and 664 residual degrees of freedom. VIF values ranged from 1.06 to 1.22, corresponding to tolerance values of approximately 0.820–0.943, providing no indication of marked multicollinearity among the included predictors. These indices assess predictor dependence and do not independently establish the adequacy of residual assumptions or external model performance.

### 3.4 Subgroup Analyses

Stratification by age category revealed elevated anxiety among younger participants (< 35 years) compared with older counterparts (≥ 45 years). In the primary adjusted model, male participants had STAI‐T scores approximately 2.8 points lower than female participants. Differences between nurses and midwives are reported by outcome in Table 3; the comparisons do not support a general claim of higher emotional burden in nurses.

**TABLE 3:** Additional exploratory comparisons between nurses and midwives.

Measure	*n*	Nurses median (IQR)	*n*	Midwives median (IQR)	Difference (95% CI)	Holm *p*
A. Continuous measures						
Trait total, rescored	720	45.0 (39.0–50.0)	177	46.0 (39.0–50.0)	−0.03 (−1.18 to 1.13)	0.731
Recalled fear	931	7.0 (5.0–10.0)	225	8.0 (5.0–10.0)	−0.48 (−1.03 to 0.06)	0.088
Recalled anxiety	930	7.0 (4.0–9.0)	224	8.0 (5.0–10.0)	−0.78 (−1.27 to −0.29)	0.006
Worry/distress	932	8.0 (5.0–10.0)	224	8.0 (5.0–10.0)	−0.32 (−0.94 to 0.30)	0.260
Recalled surprise	897	6.0 (3.0–8.0)	212	6.0 (3.0–9.0)	−0.23 (−1.01 to 0.55)	0.611
Perceived support effectiveness	767	8.0 (6.0–10.0)	193	9.0 (7.0–10.0)	−0.51 (−1.02 to −0.004)	0.046

Characteristic	Nurse *n*	%	Midwife *n*	%	Holm *p*
B. Occupational exposure and support perceptions					
Suspected‐patient contact	770	80.2	172	72.3	0.046
Diagnosed‐patient contact	736	78.0	166	70.9	0.088
Support would help: agree	572	61.2	163	71.8	0.046
Support would help: undecided	192	20.5	39	17.2	—
Support would help: disagree	171	18.3	25	11.0	—

*Note:* Continuous differences are nurse minus midwife; CIs use 20 recruitment‐unit clusters. Holm adjustment covers nine exploratory tests. Categorical valid nurse/midwife denominators are 960/238 for suspected‐patient contact, 943/234 for diagnosed‐patient contact and 935/227 for the support‐usefulness statement. The three support categories have one omnibus *p*‐value. Two dual‐role participants are excluded from these comparisons only. The additional rescored trait outcome and its valid denominators are defined in Section 2.

Abbreviations: CI, confidence interval; IQR, interquartile range.

#### 3.4.1 Additional Profession‐Specific Comparisons

Table 3 provides comparisons of 961 nurses and 238 midwives using available responses. The questionnaire item “shqetësim” is represented once as worry/distress. Support‐usefulness responses and perceived effectiveness are compared; actual access to or receipt of support cannot be compared because these outcomes are not identified in the variables used for this comparison. These direct comparisons do not substantiate a general assertion that nurses had a greater emotional burden across all measures.

Mean rescored trait totals were 44.23 in nurses and 44.25 in midwives (difference −0.03; 95% CI −1.18 to 1.13; Holm *p* = 0.731). Midwives reported higher recalled anxiety (mean 7.16 vs. 6.38; nurse‐minus‐midwife difference −0.78; 95% CI −1.27 to −0.29; Holm *p* = 0.006). Nurses more often reported suspected‐patient contact (80.2% vs. 72.3%; Holm *p* = 0.046), while agreement that support would have helped was more frequent among midwives (71.8% vs. 61.2%; omnibus Holm *p* = 0.046). These unadjusted comparisons require caution because professional roles may differ in demographics, work setting, and exposure.

### 3.5 Comparison of Included and Excluded Participants

Table 4 compares the 677 participants included in the primary regression with the 524 excluded participants. Included participants were slightly younger (38.9 ± 10.8 vs. 40.3 ± 11.0 years; *p* = 0.028; SMD = −0.13) and had less professional experience (14.0 ± 10.9 vs. 15.8 ± 11.6 years; *p* = 0.006; SMD = −0.16). Differences in female gender, nursing role, current marital status, urban residence, and chronic disease were small (absolute SMD ≤ 0.06; all *p* ≥ 0.339). Chronic disease was reported by 21.7% of included and 24.0% of excluded participants. These comparisons identify modest age and experience differences between the analytic subset and the excluded group.

**TABLE 4:** Comparison of participants included in the primary regression (*n* = 677) and excluded participants (*n* = 524).

Characteristic	Included mean (SD)	Excluded mean (SD)	SMD	*p*
A. Continuous characteristics				
Age, years	38.9 (10.8)	40.3 (11.0)	−0.13	0.028
Professional experience, years	14.0 (10.9)	15.8 (11.6)	−0.16	0.006

Characteristic	Included *n*	%	Excluded *n*	%	SMD	*p*
B. Categorical characteristics						
Female	575	84.9	448	85.5	−0.02	0.785
Nurse	541	79.9	420	80.2	−0.01	0.917
Currently married	180	26.6	140	26.7	0.00	0.960
Urban residence	610	90.1	468	89.3	0.03	0.654
Chronic disease	147	21.7	126	24.0	−0.06	0.339

*Note:* Percentages use denominators of 677 included and 524 excluded participants for each listed characteristic. Each categorical row compares the presence versus absence of that characteristic. Two‐sided *p*‐values are from Welch independent‐samples *t*‐tests for continuous variables and Pearson chi‐squared tests without continuity correction for categorical variables. No multiplicity adjustment was applied. SMDs are included minus excluded, divided by the square root of the mean of the two group variances; binary SMDs use the corresponding Bernoulli variances. Small SMDs do not establish random missingness or exclude selection on unmeasured characteristics.

Abbreviations: SD, standard deviation; SMD, standardized mean difference.

### 3.6 Model Diagnostics and Sensitivity Analyses

Table 5 summarizes model diagnostics and sensitivity analyses. Residual normality testing gave Shapiro–Wilk *W* = 0.9978 (*p* = 0.505); the studentized Breusch–Pagan statistic was *χ* ^2^(12) = 13.55 (*p* = 0.330). RESET gave *F*(2, 662) = 3.50 (*p* = 0.031), indicating possible functional‐form misspecification rather than supporting an unqualified statement that all assumptions were satisfied. Externally studentized residuals ranged from −3.21 to 3.09; three observations exceeded an absolute value of 3. Maximum leverage was 0.0488, with 10 observations above 0.0384. Maximum Cook's distance was 0.0205; 38 observations exceeded 0.00591.

**TABLE 5:** Regression diagnostics and sensitivity analyses.

Assessment	Statistic or estimate	Interpretation
Residual normality	Shapiro–Wilk *W* = 0.9978; *p* = 0.505	No clear departure detected by this test
Heteroscedasticity	Studentized Breusch–Pagan *χ* ^2^(12) = 13.55; *p* = 0.330	No clear variance departure detected
Functional form	RESET F(2, 662) = 3.50; *p* = 0.031	Possible misspecification; further functional‐form assessment is warranted
Studentized residuals	Range −3.21 to 3.09; |*r*| > 3: 3 observations	Screening, not an exclusion rule
Leverage	Maximum 0.0488; > 0.0384: 10 observations	Threshold: 2 × 13/677
Cook's distance	Maximum 0.0205; > 0.00591: 38 observations	Threshold: 4/677
HC3: fear	*B* = 1.48, 95% CI 1.28–1.68, *p* < 0.001	Positive adjusted association
HC3: worry	*B* = 0.94, 95% CI 0.73–1.15, *p* < 0.001	Positive adjusted association
HC3: support	*B* = −2.10, 95% CI −3.05 to −1.15, *p* < 0.001	Inverse adjusted association
Influence‐screening refit	*n* = 639; adjusted *R* ^2^ = 0.604	Excludes observations above the Cook's‐distance screening threshold only for sensitivity analysis
Refit: fear	*B* = 1.46, 95% CI 1.29–1.63, *p* < 0.001	Direction retained
Refit: worry	*B* = 0.96, 95% CI 0.78–1.14, *p* < 0.001	Direction retained
Refit: support	*B* = −2.11, 95% CI −2.95 to −1.27, *p* < 0.001	Direction retained
Fear‐only model	*B* = 1.51, 95% CI 1.26–1.76, *p* < 0.001; adjusted *R* ^2^ = 0.173	Unadjusted model; not interchangeable with the primary model

*Note:* The primary model retains *n* = 677. No observations were deleted from that primary analysis. HC3 does not address institution‐level dependence. A nonsignificant diagnostic test does not prove that its null assumption holds; the significant RESET result limits claims of model adequacy.

Abbreviations: *B*, unstandardized coefficient; CI, confidence interval; HC3, heteroscedasticity‐consistent covariance estimator; RESET, regression specification error test.

HC3 estimates for the principal associations were fear (*B* = 1.48, 95% CI 1.28–1.68, *p* < 0.001), worry (*B* = 0.94, 95% CI 0.73–1.15, *p* < 0.001), and reported support (*B* = −2.10, 95% CI −3.05 to −1.15, *p* < 0.001). In the influence‐screening refit (639 observations), fear (*B* = 1.46, 95% CI 1.29–1.63, *p* < 0.001), worry (*B* = 0.96, 95% CI 0.78–1.14, *p* < 0.001), and support (*B* = −2.11, 95% CI −2.95 to −1.27, *p* < 0.001) retained their directions; adjusted *R* ^2^ was 0.604. This selected‐subset fit should not be interpreted as an improvement in out‐of‐sample prediction. The fear‐only model gave *B* = 1.51, 95% CI 1.26–1.76, *p* < 0.001, with adjusted *R* ^2^ = 0.173. These checks do not resolve omitted confounding, measurement overlap, or institutional dependence.

## 4 Discussion

### 4.1 Primary Findings

In the complete‐case analytic subset, recalled pandemic‐related fear and worry were independently associated with higher STAI‐T scores after adjustment for demographic, health, occupational, and support‐related variables. Fear showed the largest standardized emotional coefficient (beta = 0.34). This pattern is consistent with reports documenting prominent fear and anxiety among nurses during COVID‐19 in other settings (Kamran, Malekpour, and Naeim 2021; Kamran, Naeim, and Rezaeisharif 2021). Because exposure and outcome information were collected cross‐sectionally in the post‐emergency period, the findings indicate association rather than causal direction.

The association of worry with STAI‐T is clinically plausible in a crisis characterized by uncertainty about infection, family risk, changing protocols, and institutional preparedness. Related work has also described broad behavioural and psychological responses to COVID‐19, reinforcing the importance of risk communication and organizational support (Kamran, Naeim, and Rezaeisharif 2020).

The inverse association of male gender with trait anxiety warrants contextual interpretation. Rather than suggesting inherent resilience, gender differences may reflect sociocultural norms in Albania where healthcare workers, predominantly female, face additional pressures including gender‐based discrimination, unequal domestic responsibilities, and societal expectations. Additionally, men may underreport emotional distress due to cultural norms discouraging emotional expression.

The strong association between chronic disease and anxiety reflects multiple psychological and physiological mechanisms. Individuals with pre‐existing chronic conditions faced elevated COVID‐19 mortality risk, creating additional personal fear. Simultaneously, chronic disease typically involves dysregulated neuroendocrine stress responses that lower thresholds for anxiety symptom emergence. Furthermore, pandemic‐related healthcare disruptions may have compromised chronic disease management, exacerbating underlying conditions and associated psychological distress.

### 4.2 Demographic and Occupational Risk Factors

Younger age emerged as a risk factor, contrary to physical COVID‐19 severity patterns. This apparent paradox may reflect differential threat perception: younger healthcare workers with less experience may have perceived the pandemic as more threatening than older colleagues with greater occupational resilience and experience navigating crises. Additionally, younger workers disproportionately reported concern about transmitting infection to family members, particularly aging parents and young children, creating secondary anxieties beyond direct personal threat.

The positive coefficient reported for work sector should be interpreted cautiously. Recruitment was anchored in participating public healthcare institutions, so this analysis does not provide a population‐representative comparison of public and private healthcare sectors. The coefficient may reflect characteristics of respondents contributing to the complete‐case model rather than a sector‐wide difference, and it should not be used to infer that public‐sector employment causes higher anxiety.

Work experience was inversely associated with trait anxiety. Potential explanations include: (1) enhanced coping skills and psychological resilience from previous crises; (2) greater occupational self‐efficacy and confidence; and (3) improved emotional regulation and stress management capabilities. This finding supports the value of mentorship programmes pairing less‐experienced with experienced colleagues during health crises.

### 4.3 The Silent Pandemic: Unmet Mental Health Needs

The finding that 80.6% of participants believed psychological support would have enhanced their pandemic coping, together with high perceived effectiveness ratings (9.0/10), indicates substantial perceived potential benefit. These perceptions support further evaluation of accessible staff‐support services but do not measure actual access, uptake, unmet service need, or treatment effectiveness.

A cross‐sectional inverse association between reported support and trait anxiety should not be interpreted as a causal protective effect. Individuals with lower anxiety may be more able to seek, receive, or report support; conversely, individuals with greater anxiety may be preferentially referred, producing confounding by indication. Service access, stigma, workplace resources, and prior mental health may influence both exposure and outcome. Perceived usefulness or expected effectiveness is distinct from actual service availability, uptake, and measured benefit. The available perceptions alone cannot establish an unmet‐service gap or treatment effectiveness.

The intolerance of uncertainty findings provide additional mechanistic insight: individuals struggling with uncertainty tolerance experienced heightened distress across all emotional domains (*r* = 0.41–0.47). This has important intervention implications—psychological approaches addressing uncertainty tolerance (e.g., Cognitive‐Behavioural Therapy for Intolerance of Uncertainty) may be particularly efficient in reducing overall emotional burden.

### 4.4 Implications and Recommendations

#### 4.4.1 Clinical and Nursing Practice

Nurse managers and occupational‐health teams should have confidential pathways for early identification of staff experiencing sustained fear, worry, or anxiety during major health emergencies, with referral options that do not depend on line‐management disclosure. Evidence from COVID‐19 nursing settings also supports structured psychological strategies, including remotely delivered cognitive‐behavioural approaches where appropriate (Naeim et al. 2021).

Institutional Planning: Nursing leadership should incorporate workforce mental‐health capacity into emergency preparedness, including protected access to psychological services, peer‐support pathways, supervisor training, workload monitoring, and clear risk communication. Practical recommendations for staff support during COVID‐19 have similarly emphasized accessible organizational responses (Kamran, Naeim, and Bagvand 2020; Naeim et al. 2020).

The intervention evidence also warrants proportional interpretation. The small quasi‐experimental virtual cognitive‐behavioural study by Naeim et al. (2021) offers preliminary support for feasibility and potential benefit, while the letters by Naeim et al. (2020) and Kamran, Naeim, and Bagvand (2020) provide practice‐oriented recommendations. Prospective evaluations with defined intervention content, timing, comparison groups, and repeated outcomes are needed before attributing anxiety reduction to a support programme.

#### 4.4.2 Education and Training

Nursing and midwifery curricula and continuing professional development should include evidence‐based stress management, communication under uncertainty, help‐seeking literacy, and team‐based crisis preparedness. These competencies are relevant not only to individual wellbeing but also to staff retention, continuity of care, and patient safety during prolonged system stress.

Research Priorities: Longitudinal studies are needed to distinguish pre‐existing trait vulnerability from crisis‐related changes and to test whether specific nursing‐led or organizational interventions improve psychological outcomes. Future analyses should also prospectively track recruitment, missing data, and intervention exposure so that selection and treatment effects can be estimated more reliably.

#### 4.4.3 Contribution to Nursing Science

By identifying fear, worry, chronic illness, perceived life‐quality deterioration, and support‐related factors within an Albanian nursing and midwifery workforce, this study adds context‐specific evidence for occupational mental‐health planning in nursing. The findings support a shift from ad hoc crisis support toward preparedness models in which psychological safety and workforce resilience are explicit components of nursing management and health‐system readiness.

### 4.5 Study Limitations

This cross‐sectional design precludes causal inference, and the post‐emergency timing means that pandemic‐specific emotional ratings were retrospective and may be affected by recall. Although stratified random selection was used within participating institutions, participation was voluntary and a centralized screening log of all invitations, refusals, and non‐returns was not maintained uniformly; selection bias therefore cannot be excluded and an exact response rate cannot be reported.

All measures were self‐reported and may be affected by social‐desirability or response bias. The single‐item 0–10 pandemic‐emotion ratings were not independently validated as a multi‐item Albanian psychometric scale. In addition, logistical distribution and collection through designated head nurses could have created perceived institutional pressure despite voluntary participation and anonymity.

The primary multivariable analysis was a complete‐case analysis of 677 of 1201 participants (56.4%); 524 participants (43.6%) lacked at least one variable required for that model and were not included. No imputation was performed, and the missingness mechanism cannot be assumed to be random. The regression estimates may therefore be affected by complete‐case selection and should not be generalized uncritically to the full descriptive cohort. Generalizability beyond the participating public institutions, particularly to private‐sector or remote settings, also remains uncertain.

## 5 Conclusion

This study provides evidence that recalled pandemic‐related fear and worry were independently associated with trait anxiety in the complete‐case subset of Albanian nurses and midwives. Chronic disease and worsened perceived life quality were also associated with higher STAI‐T scores, whereas older age, greater professional experience, male gender, and reported psychological support showed inverse associations. These findings should be interpreted as cross‐sectional associations rather than causal effects.

The high proportion of participants who believed psychological support would have helped them cope better indicates a substantial perceived support need. The complete‐case analysis further suggests that psychological support deserves prospective evaluation as a potentially modifiable organizational factor; the present observational design does not establish intervention effectiveness.

For nursing services, the practical priority is to integrate confidential psychological support, workforce monitoring, supervisor preparedness, and resilience‐building into routine emergency planning. Prospective longitudinal and intervention studies are needed to determine which approaches produce durable benefits for nurses, midwives, and the systems in which they deliver care.

### 5.1 Relevance to Nursing Practice and Science

For nursing practice, these findings identify staff fear, worry, chronic illness, and perceived deterioration in life quality as signals that can inform confidential workforce‐support pathways during health emergencies. For nursing management, they support preparedness plans that combine clear risk communication, access to psychological services, peer and supervisory support, and special attention to less‐experienced staff. For nursing science, the Albanian data extend evidence from larger health systems and highlight the need for longitudinal evaluation of organizational interventions rather than relying on cross‐sectional associations alone.

## Author Contributions

**Anila Cake:** conceptualization, methodology, project administration, writing – original draft. **Ergys Ramosaçaj:** supervision, data curation, methodology, writing – review and editing. **Artan Simaku:** formal analysis, validation, writing – review and editing, software. **Agron Bytyqi:** formal analysis, writing – review and editing, writing – original draft, visualization. **Flora Zyberaj:** investigation, writing – review and editing.

## Funding

The authors have nothing to report.

## Disclosure

Patient or Public Contribution: Participants were nurses and midwives. No patient or public contribution to the research process was reported.

Reporting Guideline: The study is reported in accordance with the STROBE recommendations for cross‐sectional observational studies. The completed checklist is provided as Supplementary File 1.

## Ethics Statement

The study received ethical approval from the Ethics Council of the University of Medicine, Tirana, Albania (protocol no. 825/2, decision no. 10, March 18, 2024), and authorizations from 17 university, regional, and municipal hospitals across Albania. All participants provided informed consent, and confidentiality and anonymity were strictly maintained.

## Consent

No identifiable individual participant information is reported.

## Conflicts of Interest

The authors declare no conflicts of interest.

## Supporting information


**Supplementary File 1:** STROBE checklist: cross‐sectional study.

## Data Availability

The study data are not publicly available because of ethical and confidentiality restrictions. Requests for de‐identified data may be addressed to the corresponding author and are subject to institutional and ethical requirements.
