# Supplementary material for: Psychological Impact of COVID‐19 on Nurses and Midwives in Albania: Fear, Anxiety and Predictors of Emotional Burden
Source: Nurs Open. 2026 Nov 7;13(11):e71001. doi: 10.1002/nop2.71001 (PMC13744009; doi:10.1002/nop2.71001)
Supplement: Supplementary file 1 [file NOP2-13-e71001-s001.docx]

Supplementary File 1

# STROBE checklist: cross-sectional study

Manuscript 8888600: Psychological Impact of COVID-19 on Nurses and Midwives in Albania: Fear, Anxiety, and Predictors of Emotional Burden. Locations refer to the 21-page highlighted manuscript with continuous line numbering. Tables 1–5 are supplied in the separate Tables file; funding is reported on page 2 of the separate title page.

Adapted from the STROBE Initiative cross-sectional checklist; item labels are abbreviated. Source: https://www.strobe-statement.org/checklists/ (accessed 10 October 2026).

| Item | Element | Location and reporting |
| --- | --- | --- |
| 1a | Design | p. 1, lines 5–20; p. 3, lines 70–75. The cross-sectional design is identified. |
| 1b | Abstract | p. 1, lines 5–20. Design, setting, sample, descriptive and adjusted results, and complete-case scope are summarized. |
| 2 | Rationale | pp. 1–3, lines 24–67. Background, nursing literature, intervention context and rationale are presented. |
| 3 | Objectives | p. 3, lines 64–67. Four study objectives are stated. |
| 4 | Design | p. 3, lines 70–75; p. 3, lines 76–80. Cross-sectional design and retrospective pandemic ratings are explained. |
| 5 | Setting/dates | p. 3, lines 70–75. March 2024–May 2025; 17 public healthcare institutions in Albania. |
| 6a | Selection | p. 4, lines 84–104. Sampling frame, stratification, random selection, replacement, and eligibility criteria. |
| 7 | Variables | pp. 5–6, lines 110–138; p. 7, lines 163–167; p. 7, lines 168–176; Table 2. Measures, the primary predictor set, continuous and indicator coding, reference categories and entry method are described. |
| 8 | Measurement | pp. 4–6, lines 106–144; p. 8, lines 188–193. Questionnaire administration, instruments, score ranges and profession-comparison scoring; measurement-equivalence limitations are acknowledged. |
| 9 | Bias | p. 6, lines 145–149; p. 17, lines 435–450. Anonymity, recall, reporting, selection and common-method bias are addressed. |
| 10 | Study size | p. 4, lines 95–99. Sample-size assumptions and achieved descriptive cohort of 1,201. |
| 11 | Quantitative variables | pp. 5–8, lines 113–199; Tables 1–5. Scale ranges, descriptive summaries and comparison methods are provided. Reasons for all category boundaries are not reported. |
| 12a | Statistical methods | pp. 6–8, lines 155–215; p. 11, lines 289–293; Table 5. Statistical methods, covariates, VIF/tolerance, residual and influence diagnostics, sensitivity analyses and limitations are described. |
| 12b | Subgroups | pp. 6–7, lines 159–162; p. 8, lines 188–193; p. 8, lines 194–199. Group tests and exploratory nurse–midwife comparisons with Holm adjustment; no interaction analyses are reported. |
| 12c | Missing data | p. 6, lines 150–153; p. 6, lines 155–158; p. 7, lines 177–180; p. 8, lines 188–193. Valid-response denominators, complete-case regression, no imputation, and treatment of invalid trait-item responses. |
| 12d | Sampling analysis | p. 4, lines 88–94; p. 8, lines 194–215; p. 13, lines 331–338. Stratified recruitment and clustered confidence intervals for profession mean differences are described. Design-based adjustment for the primary regression is not reported. |
| 12e | Sensitivity | p. 8, lines 207–215; p. 13, lines 331–338; Table 5. HC3, influence-screening and fear-only sensitivity analyses are described. |
| 13a | Participant flow | p. 10, lines 242–245; p. 4, lines 95–99. Descriptive cohort: 1,201; primary regression: 677; excluded from that regression: 524. Earlier invitation and screening totals are unavailable. |
| 13b | Nonparticipation | p. 4, lines 95–99; p. 10, lines 242–245. Recruitment logs were not centralized; an exact response rate cannot be given. Regression exclusion reflects incomplete required variables. |
| 13c | Flow display | p. 10, lines 242–245. Participant numbers are presented in prose; no flow diagram is included. |
| 14a | Characteristics | p. 9, lines 235–241; p. 12, lines 302–306; p. 12, lines 308–314; pp. 12–13, lines 316–322; Tables 1, 3 and 4. Overall characteristics, profession comparisons and included/excluded comparison. |
| 14b | Missingness | p. 6, lines 155–158; Tables 3–4 and their notes. Valid denominators are supplied for additional comparisons. A complete variable-by-variable missing-data table for all primary-model variables is not provided. |
| 15 | Outcome | p. 10, lines 247–257; p. 12, lines 308–314; Table 3. Emotional-item summaries and profession-specific rescored trait summaries; a full-cohort STAI-T summary is not separately reported. |
| 16a | Estimates | pp. 10–11, lines 262–293; Table 2. Adjusted coefficients and 95% confidence intervals. Tables 3–4 report unadjusted group comparisons; corresponding unadjusted coefficients for every primary-model predictor are not provided. |
| 16b | Categories | p. 9, lines 235–241; Table 1. Age and professional-experience categories are presented. |
| 16c | Absolute risk | Continuous trait-anxiety outcome; relative-to-absolute risk conversion does not apply. |
| 17 | Other analyses | p. 12, lines 302–306; p. 12, lines 308–314; pp. 12–13, lines 316–322; p. 13, lines 331–338; Tables 3–5. Profession, included/excluded and sensitivity comparisons are reported. |
| 18 | Key findings | p. 13, lines 341–347; p. 18, lines 452–466. Main associations are summarized in relation to the objectives. |
| 19 | Limitations | p. 17, lines 435–450. Design, recall, self-report, measurement and complete-case selection limitations are acknowledged. Recall-bias direction and magnitude are discussed on p. 6, lines 145–149; model qualifications are reported on p. 8, lines 200–215, and p. 13, lines 324–338. |
| 20 | Interpretation | pp. 13–18, lines 341–466. Findings are related to previous literature, with explicit cross-sectional and noncausal qualifications. |
| 21 | Generalizability | pp. 12–13, lines 316–322; p. 17, lines 445–450; Table 4. Observed included/excluded differences and limits on generalizing the complete-case estimates to the full cohort and beyond participating public institutions. |
| 22 | Funding | Separate title page, p. 2, Funding. The authors report no external financial support for this study. |
